# 

**Published:** 1909-09-15

**Authors:** 


### ANNOUNCEMENTS.

SOUTHWESTERN MICHIGAN DENTAL SOCIETY elected the following officers for the ensuing year: President A. A. Welch, Battle Creek; Vice President, R. A. Bowie, Three Rivers; Secretary-Treasurer, C. W. Johnson, Lawton. Next place of meeting Coldwater, Mich., second Tuesday and Wednesday in April, 1910.

THE OHIO STATE DENTAL BOARD will hold its regular fall meeting in Columbus on October 19-22, 1909, for the examination of applicants for license. All applications should be in the hands of the secretary not later than Oct. 9th, together with the fee of $25.00.

For further information and blank applications address

F. R. CHAPMAN, *Sec'y*,
305 Schultz Bldg.,
Columbus, O.

